# *HMGA1* pseudogenes as candidate proto-oncogenic competitive endogenous RNAs

**DOI:** 10.18632/oncotarget.2202

**Published:** 2014-07-15

**Authors:** Francesco Esposito, Marco De Martino, Maria Grazia Petti, Floriana Forzati, Mara Tornincasa, Antonella Federico, Claudio Arra, Giovanna Maria Pierantoni, Alfredo Fusco

**Affiliations:** ^1^ Istituto di Endocrinologia ed Oncologia Sperimentale del CNR c/o Dipartimento di Medicina Molecolare e Biotecnologie Mediche, Scuola di Medicina e Chirurgia di Napoli, Università degli Studi di Napoli “Federico II”, Naples, Italy.; ^2^ Istituto Nazionale dei Tumori, Fondazione Pascale, Naples, Italy.

**Keywords:** HMGA1P6, HMGA1P7, HMGA1, ceRNA, miRNA

## Abstract

The High Mobility Group A (HMGA) are nuclear proteins that participate in the organization of nucleoprotein complexes involved in chromatin structure, replication and gene transcription. HMGA overexpression is a feature of human cancer and plays a causal role in cell transformation. Since non-coding RNAs and pseudogenes are now recognized to be important in physiology and disease, we investigated *HMGA1* pseudogenes in cancer settings using bioinformatics analysis. Here we report the identification and characterization of two *HMGA1* non-coding pseudogenes, *HMGA1P6* and *HMGA1P7*. We show that their overexpression increases the levels of HMGA1 and other cancer-related proteins by inhibiting the suppression of their synthesis mediated by microRNAs. Consistently, embryonic fibroblasts from *HMGA1P7*-overexpressing transgenic mice displayed a higher growth rate and reduced susceptibility to senescence. Moreover, *HMGA1P6* and *HMGA1P7* were overexpressed in human anaplastic thyroid carcinomas, which are highly aggressive, but not in differentiated papillary carcinomas, which are less aggressive. Lastly, the expression of the *HMGA1* pseudogenes was significantly correlated with HMGA1 protein levels thereby implicating *HMGA1P* overexpression in cancer progression. In conclusion, *HMGA1P6* and *HMGA1P7* are potential proto-oncogenic competitive endogenous RNAs.

## INTRODUCTION

The High-Mobility Group A (HMGA) family consists of three proteins: HMGA1a, HMGA1b, and HMGA2 [1]. HMGA proteins do not have transcriptional activity *per se*; however, by interacting with the transcription machinery, they alter the chromatin structure and thereby regulate the transcriptional activity of various genes [2, 3]. The levels of HMGA proteins are low or absent in normal cells and adult tissues [4]. In contrast, their constitutive expression is remarkably high in neoplastically transformed cells and in embryonic cells [5-7]. Their overexpression is associated with a highly malignant phenotype and correlates with the presence of metastasis and reduced survival [8, 9]. Several studies implicate the expression of the HMGA genes in the process of carcinogenesis [10-18]. However, although HMGA overexpression is known to play a critical role in malignant cell transformation, the mechanisms regulating HMGA protein levels remain largely obscure.

Non-coding RNAs, including pseudogenes, have long been viewed as non-functional genomic relicts of evolution, but a large body of evidence now suggests they are important in both physiology and disease. Pseudogenes are usually defined as defunct copies of genes that have lost their potential as DNA templates for functional products [19-26] because they harbour premature or delayed stop codons, deletions/insertions and frameshift mutations that abrogate their translation into functional proteins. There are two types of pseudogenes: processed pseudogenes, which have been retrotransposed back into a genome *via* an RNA intermediate; and nonprocessed pseudogenes, which are the genomic remnants of duplicated genes or residues of dead genes. Processed pseudogenes contain no introns, and share 5' and 3' untranslated region (UTR) sequences with their ancestral genes [27]. Since miRNAs repress target gene expression by binding to complementary sequences in the 3' UTR of target mRNA, pseudogenes can be targeted by miRNAs that modulate the expression of coding genes. Indeed, several pseudogene transcripts exert regulatory control of their ancestral gene expression levels by competing for the same miRNAs [28], which is in keeping with the notion that miRNA activity is theoretically affected by the availability of target microRNA response elements (MRE) in the cellular milieu [28-30]. Given this scenario, we studied the possible functional relationship between the mRNAs produced by the *HMGA1* oncogene and its pseudogenes (*HMGA1Ps*), and the consequences of this interaction especially in the process of carcinogenesis in which HMGA1 overexpression plays a critical role.

## RESULTS

### *HMGA1P6* and *HMGA1P7* are targeted by *HMGA1*-targeting miRNAs

**Figure 1:**
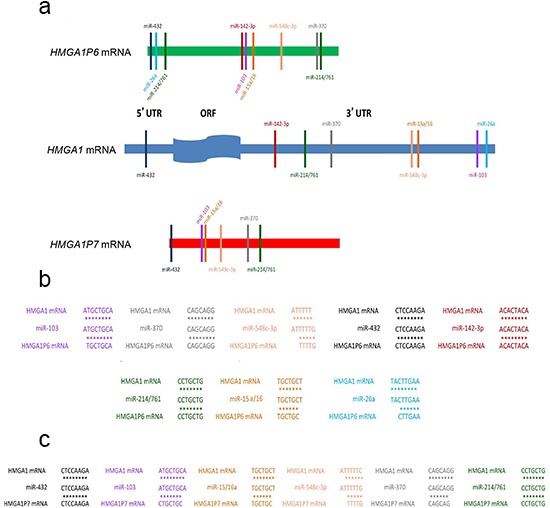
*HMGA1P6* and *HMGA1P7* show the same seed sequences of *HMGA1*-targeting miRNAs **(A)** *HMGA1* (middle), *HMGA1P6* (top) and *HMGA1P7* (bottom) UTRs contain highly conserved regions. HMGA1-targeting miRNA seed matches within the high homology region are conserved between *HMGA1* and *HMGA1Ps*. **(B)** and **(C)** binding of HMGA1-targeting miRNAs to *HMGA1P6* (B) and *HMGA1P7* (C).

We first identified seven *HMGA1Ps* by bioinformatics analysis. Of these, we focused on the *HMGA1P6* and *HMGA1P7* processed pseudogenes located at 13q12.12 and 6q23.2, respectively, because of their very high sequence homology with *HMGA1* in the coding region and in the 5' and 3' UTRs (Figure 1A). A missense mutation of the initiator methionine codon prevents translation of *HMGA1P7* whereas *HMGA1P6* is non-protein coding since it carries a mutation in the stop codon and so generates a non-translatable mRNA. Within the high homology regions, we found perfectly conserved seed matches for miRNAs that have been predicted (miR-103, miR-142-3p, miR-370, and miR-432) or already demonstrated (miR-15 [31], miR-16 [31], miR-26a [32], miR-214 [33], miR-548c-3p [34] and miR-761 [33]) to target the *HMGA1* gene (Figure 1B and 1C).

**Figure 2:**
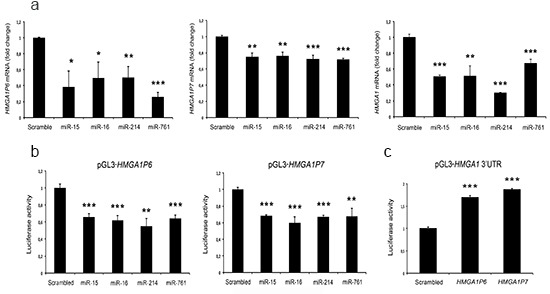
*HMGA1P6* and *HMGA1P7* are targeted by *HMGA1*-targeting miRNAs **(A)** qRT-PCR analysis of *HMGA1P6* (left), *HMGA1P7* (middle) and *HMGA1* (right) mRNA from the MCF7 cells transfected with scrambled-oligonucleotide, miR-15, miR-16, miR-214 and miR-761.**(B)** *HMGA1Ps* were cloned into the pGL3 control vector. Relative luciferase activity in HEK293 cells transiently transfected with miR-15, miR-16, miR-214, miR-761 and a control scrambled oligonucleotide. **(C)** The 3'UTR of *HMGA1* was cloned into the pGL3 control vector. Relative luciferase activity in HEK293 cells transiently transfected with the empty vector, *HMGA1P6* and *HMGA1P7*.. The results are reported as the mean of values. Error bars represent mean ± SD; n=3. *, *P* < 0.05 **, *P* < 0.01 ***, *P* < 0.001 (*t* test).

To evaluate the ability of these miRNAs to target *HMGA1P6* and *HMGA1P7*, we transfected miR-15, miR-16, miR-214 and miR-761 into MCF7 cells (human breast adenocarcinoma), and measured *HMGA1P6*, *HMGA1P7*, and *HMGA1* mRNA levels using Real-time PCR and PCR primer sets (see Methods) that discriminate the three mRNA transcripts. As shown in Figure 2A, the transfection of the *HMGA1*-targeting miRNAs led to a significant reduction of *HMGA1*, *HMGA1P6* and *HMGA1P7* mRNA levels.

To determine whether the *HMGA1*-targeting miRNAs directly interacted with the *HMGA1P* mRNAs, we inserted the full-length *HMGA1P6* and *HMGA1P7* mRNAs downstream of the luciferase open reading frame. These reporter vectors were transfected into human embryonic kidney (HEK293) cells together with miRNA precursors and a control non-targeting scrambled oligonucleotide. The luciferase activity was much lower after miR-15, miR-16, miR-214 and miR-761 transfection compared with the scrambled oligonucleotide (Figure 2B). These results indicate that the *HMGA1Ps* and *HMGA1* undergo the same miRNA-mediated post-transcriptional regulation.

### *HMGA1P6* and *HMGA1P7* act as decoys for *HMGA1*-targeting miRNAs

**Figure 3:**
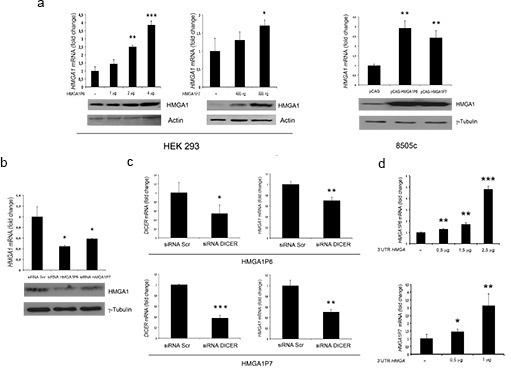
HMGA1 is positively regulated by *HMGA1Ps* **(A**) (upper panels) qRT-PCR analysis of *HMGA1* mRNA levels in HEK293 and 8505c cells transfected with the empty vector, *HMGA1P6* and *HMGA1P7*. (Lower panels) Western blot analysis of HMGA1 protein levels from the same samples shown in the upper panels. **(B**) (Upper panel) qRT-PCR analysis of *HMGA1* mRNA levels in 8505c cells transfected with the scrambled oligonucleotide, siRNA-*HMGA1P6* and siRNA-*HMGA1P7*s. (Lower panel) Western blot analysis of HMGA1 protein levels from the same samples shown in the upper panel. **(C)** *HMGA1* mRNA levels 24 h after the transfection of *HMGA1P6* and *HMGA1P7* in scrambled oligonucleotide or siRNA-*DICER* 8505c transfected cells. **(D)** *HMGA1P6* and *HMGA1P7* mRNA levels after the transfection of the 3'UTR of the *HMGA1* plasmids in MCF7 cells. The results are reported as the mean of values; Error bars represent means ± SD; n =3. *, *P* < 0.05 **, *P* < 0.01 ***, *P* < 0.001 (*t* test).

Subsequently, we examined the ability of *HMGA1P6* and *HMGA1P7* to function as a decoy for *HMGA1*-targeting miRNAs using a vector carrying the 3' UTR of the *HMGA1* mRNA downstream of the luciferase open reading frame. This reporter vector was transfected into HEK293 cells together with *HMGA1P6*- or *HMGA1P7*-expressing vectors. As expected, luciferase activity was much higher in *HMGA1Ps-*transfected cells than in the control vector (Figure 2C). Moreover, overexpression of different amounts of *HMGA1P6* or *HMGA1P7* drastically and dose-dependently reduced the effects exerted by miRNA on the levels of both the *HMGA1* transcript and protein (Figure 3A). Conversely, *HMGA1P6* and *HMGA1P7* knockdown resulted in decreased *HMGA1* mRNA and protein levels (Figure 3B) thereby mirroring the results obtained with *HMGA1P6* and *HMGA1P7* overexpression (Figure 3A). Therefore, the *HMGA1Ps* compete for the endogenous miRNA-binding sites.

The upregulation of HMGA1 induced by overexpression of *HMGA1P6* and *HMGA1P7* was blunted in DICER-silenced cells (Figure 3C). In fact, silencing of DICER, the enzyme that catalyses the last step of miRNA maturation, leads to reduced levels of mature miRNAs compared to control cells. These results support the notion that *HMGA1P6* and *HMGA1P7* require mature miRNAs to regulate HMGA1 levels. Finally, as expected from our observation that *HMGA1Ps* increase HMGA1 levels, we found that the *HMGA1* 3' UTR upregulates *HMGA1P* levels (Figure 3D).

### *HMGA1P6* and *HMGA1P7* exert oncogenic activity

The *HMGA1* pseudogenes can be transcribed but they cannot code for protein. However, the above-reported results suggest that they derepress *HMGA1* transcript and protein levels (Figure 3A). To evaluate the functional consequences of *HMGA1P6* and *HMGA1P7* overexpression, we investigated their role in cellular proliferation, apoptosis, migration and invasion in cells expressing HMGA1.

**Figure 4:**
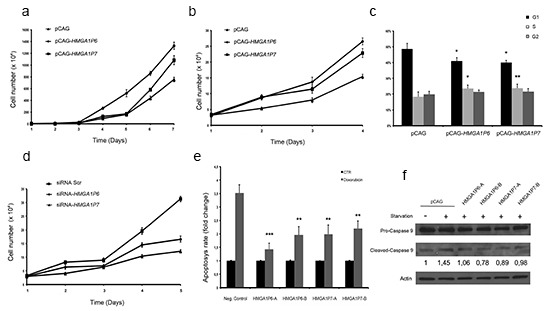
*HMGA1P6* and *HMGA1P7* expression increases cell proliferation and reduces apoptosis **(A)** and **(B)** HEK293 and 8505c cell proliferation in *HMGA1P6*- and *HMGA1P7*-transfected cells. **(C)** HEK293 cells were transfected with the control, *HMGA1P6* or *HMGA1P7* vectors. The DNA content of the transfected HEK293 cells was analyzed by flow cytometry after propidium iodine staining. **(D)** 8505c cell proliferation in siRNA-*HMGA1P6*- and siRNA-*HMGA1P7*-transfected cells. **(E)** *HMGA1P6*- and *HMGA1P7*-transfected cells were treated with doxorubicin, and apoptosis was assessed by FACS. **(F)** HEK293 cells were starved, and apoptosis was assessed by Western blot analysis of Caspase 9 cleavage. The results are reported as the mean of values; Error bars represent means ± SD; n =3. *, *P* < 0.05 **, *P* < 0.01 ***, *P* < 0.001 (*t* test).

As shown in Figure 4A and B, HEK293 cells and 8505c cells (derived from a human anaplastic thyroid carcinoma) transfected with *HMGA1P6*- or *HMGA1P7*-expressing vectors grew significantly faster than the empty vector-transfected cells. Cell cycle analysis of the cells overexpressing *HMGA1P6* and *HMGA1P7* revealed an increased number of cells in the S phase and a reduced number of cells in G1 compared with control cells (Figure 4C). This was not unexpected given the increased HMGA1 levels induced by *HMGA1P6* and *HMGA1P7* expression. Moreover, in 8505c cells knocked down for the *HMGA1Ps*, we found that 8505c-siRNA-*HMGA1P6* and 8505c-siRNA-*HMGA1P7* cells grew at a significantly slower rate than the 8505c-siRNA negative control (Figure 4D). Interestingly, cell cycle analysis of the 8505C-siRNA-*HMGA1P6* and 8505C-siRNA-*HMGA1P7* cells revealed an increase in the number of cells in the sub-G1 phase, which corresponds to apoptotic cells, compared with control cells (data not shown). This result is in agreement with the finding that HMGA silencing induces apoptosis in cancer cells [12].

To probe further the role of *HMGA1* pseudogenes in apoptotic cell death, we incubated HEK293 cells with doxorubicin in the presence or absence of the *HMGA1Ps*. As shown in Figure 4E, *HMGA1P6* and *HMGA1P7* overexpression significantly reduced the programmed cell death induced by doxorubicin. The same result was obtained with HEK293 cells in which apoptosis was induced by serum-starvation. Indeed, the overexpression of the *HMGA1Ps* counteracted caspase 9 cleavage (Figure 4F).

**Figure 5:**
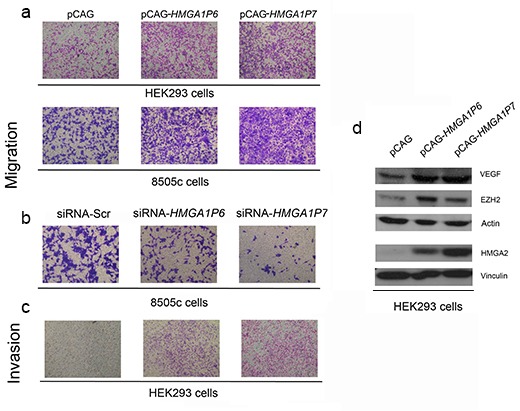
The expression of the *HMGA1Ps* affects cell migration and invasion **(A**) Cell migration assays of HEK293 and 8505c cells transfected with *HMGA1P6* or *HMGA1P7* or with a control vector. One representative experiment is reported. **(B**) Cell migration assays of 8505c cells transfected with siRNA-*HMGA1P6* or siRNA-*HMGA1P7* or with a empty vector. One representative experiment is reported. **(C)** Cell invasion assays of HEK293 cells transfected with *HMGA1Ps* or with the backbone vector. One representative experiment is reported. **(D)** Extracts from HEK293 transfected with *HMGA1P6* or *HMGA1P7* or with a control vector were analyzed by Western blotting.

Since HMGA1 promotes cell migration and invasion [8] we carried out cell migration and invasion assays in cells transfected with the *HMGA1Ps*. As expected, cell migration was significantly higher in HEK293 and 8505c cells overexpressing *HMGA1P6* or *HMGA1P7* than in control cells (Figure 5A). Moreover, 8505c-siRNA-*HMGA1P6* and 8505c-siRNA-*HMGA1P7* cells migrated more slowly than the 8505c-siRNA negative control (Figure 5B). Accordingly, the invasion matrigel assay revealed invasion activity in HEK293 cells transfected with *HMGA1P6* or *HMGA1P6* (Figure 5C). Similar results were obtained with the *HMGA1Ps*-8505c cells (data not shown). These results indicate that cell proliferation, motility and invasion is driven by regulation *HMGA1Ps*-mediated of *HMGA1*.

Bioinformatic analysis revealed that *HMGA1P6* and *HMGA1P7* contain sequences that can be targeted by miRNAs that target *High Mobility Group A2 (HMGA2)*, *Vascular Endothelial Growth Factor (VEGF)* and *Enhancer of Zeste Homolog 2 (EZH2)*, all of which are known to be involved in carcinogenesis [34-36]. Accordingly, we found that *HMGA1P6* or *HMGA1P7* overexpression increased the level of the proteins coded for by these genes (Figure 5D). Consequently, it appears that *HMGA1P6* and *HMGA1P7* expression may contribute to cancer progression by acting as decoys for cancer-related genes other than *HMGA1*.

### Correlation of HMGA1 and the overexpression of the *HMGA1Ps* in human cancer

**Figure 6:**
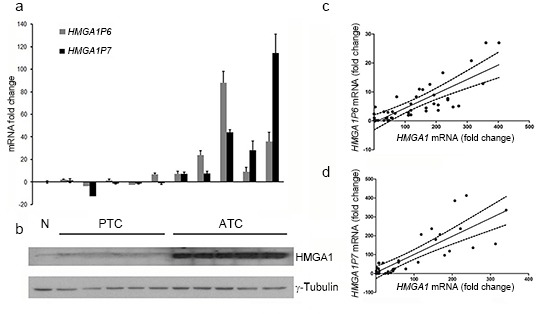
HMGA1 protein expression positively correlates with the expression of the *HMGA1Ps* in ATC **(A)** *HMGA1P6* and *HMGA1P7* qRT-PCR analysis in normal thyroid tissue (NT), papillary thyroid carcinoma (PTC) and anaplastic thyroid carcinoma (ATC). The results are reported as the mean of expression values. The error bars represent mean ± SD; n = 3. **(B)** Western blot analysis of HMGA1 protein expression in the same samples as in A. **(C)** and **(D)** Ovarian sample expression values derived from commercial sources were combined for correlation analysis. Linear regressions of *HMGA1* versus *HMGA1P6* (C) and *HMGA1* versus *HMGA1P7* (D) are shown.

To verify whether the two *HMGA1Ps* function as decoys in the regulation of HMGA1 protein levels also in human cancer, we analyzed the expression of HMGA1 and of the *HMGA1Ps* in a panel of differentiated and undifferentiated thyroid carcinomas by Western Blotting and Real-time PCR. As shown in Figure 6, papillary (PTC) thyroid carcinomas, which are well differentiated and poorly aggressive, expressed low levels of *HMGA1P6* and *HMGA1P7* (Figure 6A). Conversely, anaplastic thyroid carcinoma (ATC), which is one of the most aggressive human tumours, expressed very high *HMGA1P* levels that, moreover, correlated with HMGA1 protein levels (Figure 6B). Accordingly, HMGA1 expression, which was undetectable in normal thyroid tissue, was much higher in ATC than in PTC. Similar results were obtained in human ovarian carcinomas (see Figure 6C and 6D). The direct correlation between *HMGA1* and *HMGA1P6* expression (r=0.6553, P<0.0001) and between *HMGA1* and *HMGA1P7* expression (r=0.7001, P<0.0001) suggests that these genes are co-regulated (Figure 6C and 6D). Taken together, these results indicate that *HMGA1P6, HMGA1P7* and *HMGA1* expression is correlated with cancer aggressiveness.

### *HMGA1P7* overexpressing mouse embryonic fibroblasts grow faster and senesce later

**Figure 7:**
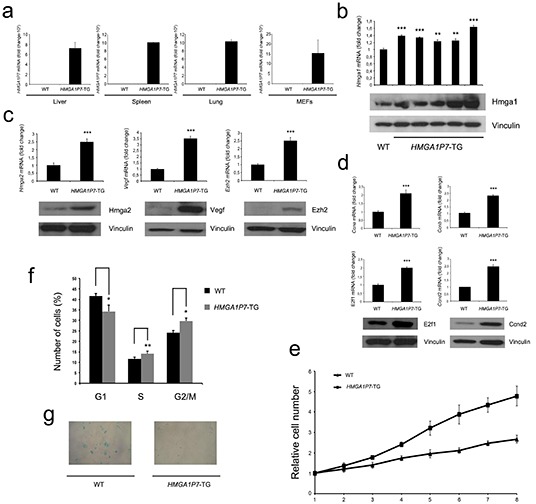
*HMGA1P7* overexpressing MEFs show a higher growth rate, and lower susceptibility to senescence **(A)** qRT-PCR analysis of total RNA from livers, spleens, lungs, and MEFs of WT and *HMGA1P7* transgenic mice. **(B)** (upper panel) qRT-PCR analysis of *HMGA1* mRNA levels in WT and *HMGA1P7* transgenic MEFs. (Lower panel) Western blot analysis of HMGA1 protein expression in the same samples. **(C)** and **(D)** qRT-PCR and Western blot analysis of genes that share common miRNAs with *HMGA1* (Left panel) and *HMGA1*-regulated genes (Right panel). **(E)** MEFs were prepared from WT and *HMGA1P7* overexpressing embryos at 12.5 dpc. At culture passage 3, they were plated and counted daily for 8 days. **(F)** Propidium iodide flow cytometry of asynchronous growing WT and *HMGA1P7* overexpressing MEFs. **(G)** Light microscopy of representative WT and *HMGA1P7* overexpressing MEFs stained for β-galactosidase activity at culture passages 6. The results are reported as the mean of values with *error bars* indicating SD (mean ± SD); n =3. *, *P* < 0.05 **, *P* < 0.01 ***, *P* < 0.001 (*t* test).

To establish the role of the *HMGA1Ps in vivo*, we generated transgenic mice overexpressing *HMGA1P7*. The expression of *HMGA1P7* in transgenic mice was verified by RT-PCR using RNAs extracted from liver, spleen, lung and mouse embryonic fibroblasts (MEFs). *HMGA1P7* mRNA levels were high in the tissues of *HMGA1P7* transgenic mice and absent in the WT counterpart (Figure 7A). Consistently, *HMGA1* transcript and protein levels were significantly higher in *HMGA1P7*-MEFs than in the WT control (Figure 7B). Notably, *HMGA1P7*-MEFs also expressed increased levels of the Hmga2, Ezh2 and Vegf proteins, which are coded for by genes that share miRNAs with *HMGA1* (Figure 7C), and increased levels of the HMGA1-regulated genes *Ccna*, *Ccnb*, *Ccnd2* and *E2f-*1 (Figure 7D), which play a critical role in cell cycle regulation [8].

As expected from the high HMGA1 expression in the *HMGA1P7*-MEFs, the growth rate of these MEFs was significantly higher than that of the WT controls (Figure 7E). To determine whether the higher growth rate of *HMGA1P7*-MEFs was caused by altered progression through the cell cycle, we examined asynchronously growing MEFs by flow cytometry. The number of *HMGA1P7-*MEFs was lower in G1 and higher in the S phase of the cell cycle compared with WT MEFs (Figure 7F).

We, next, examined the susceptibility of MEFs to senescence by measuring senescence-associated β-gal (SA-β-gal) activity. At culture passage 6, SA-β-gal activity was present in WT MEFs, but not in the *HMGA1P7* transgenic counterparts (Figure 7G). These findings indicate that *HMGA1P7* overexpression reduced susceptibility to cellular senescence.

### Identification of the genes modulated by *HMGA1P7* expression

To identify the genes regulated by *HMGA1P7* expression, we analyzed the expression profile of WT and *HMGA1P7* transgenic MEFs in microarray analyses. To this aim, RNAs extracted from WT and *HMGA1P7*-MEFs were hybridized to the Affymetrix GeneChip Mouse Gene 2.0 ST oligonucleotide arrays. Seventy transcripts that had a significant fold change variation (p <0.05) were examined as candidate genes involved in *HMGA1P7* tumour-promoting activity. Interestingly, we found five upregulated cancer-related genes (*Epha3, Hjurp, Kif26, S1pr3* and *Pde3B*) that shared miRNAs with the *HMGA1P7* transcript. These genes are involved in various human cancers (glioblastoma, breast and hematological cancers), and are candidate therapeutic targets [37-41]. Real-time PCR experiments confirmed upregulation of all these genes in *HMGA1P7-*MEFs (Supplementary Figure 1). These results support the concept that *HMGA1P7* modulates the expression of several cancer-related genes by acting as a ceRNA.

## DISCUSSION

The HMGA proteins play a critical role in carcinogenesis. Recently, several miRNAs have been demonstrated to target these genes [31-34], and their dysregulation may contribute to HMGA1 protein overexpression in human neoplasias [31, 32, 34]. Moreover, an important role in the regulation of protein synthesis has recently been ascribed to pseudogenes [27, 28]: the presence of the same miRNA-targeted seed sequences in the *HMGA1* and in the *HMGA1Ps* UTR regions could block the access of miRNAs to their protein-coding target genes. Finally, it has been outlined a novel gene-expression pathway in which an *HMGA* protein-coding gene, *Hmga2*, operates largely independently of its protein-coding function to promote cancer progression as a competing endogenous RNA [42].

We asked whether *HMGA1* pseudogenes affect HMGA1 protein levels, and, consequently, whether they play a critical role in cancer progression. We focused on *HMGA1P*6 and *HMGA1P*7, which have conserved seed matches for miRNAs targeting the *HMGA1* gene in the high homology regions. We demonstrate that overexpression of these *HMGA1* pseudogenes increases HMGA1 protein levels, and inhibits the suppression of HMGA1 protein synthesis by miRNAs that target the *HMGA1* gene, namely, miR-15, miR-16, miR-214, and miR-761 [31-34]. Consistent with these results, our functional studies demonstrate that *HMGA1P*6 and *HMGA1P*7 overexpression increases the cell growth rate by decreasing the number of G1-phase cells and increasing the number of S-phase cells, compared with the backbone vector-transfected cells. Therefore, *HMGA1P*6 and *HMGA1P*7 affect cell cycle progression, as expected, given their ability to increase the protein levels of HMGA1, which is involved in the regulation of the G1-S transition phase of the cell cycle [34]. Moreover, *HMGA1P*6 and *HMGA1P*7 overexpression increased cell migration and invasiveness, and decreased the apoptotic rate.

These results prompted us to verify whether *HMGA1P*6 and *HMGA1P*7 overexpression is involved also in human carcinogenesis. Interestingly, *HMGA1P*6 and *HMGA1P*7 were abundantly expressed in ATC, which are very aggressive and express very high HMGA1 protein levels [43]. Conversely, *HMGA1P*6 and *HMGA1P*7 expression was low in PTC, which are well differentiated and poorly aggressive, and express moderate HMGA1 protein levels. We obtained similar results in human ovarian carcinomas suggesting that *HMGA1Ps* can regulate HMGA1 protein levels also *in vivo*.

Interestingly, *HMGA1P6* and *HMGA1P7* seem to affect cancer progression also by binding to the same miRNAs that target proteins involved in cancer progression. Indeed, the overexpression of the *HMGA1Ps* increased also the levels of HMGA2, VEGF and EZH2 that are coded for by genes targeted by *HMGA1*-targeting miRNAs. Notably EZH2, which is involved in carcinogenesis, is overexpressed in ATC but not in PTC [44].

Data obtained with transgenic mice overexpressing *HMGA1P7* and with the relative MEFs support the concept that *HMGA1P7* plays an oncogenic role. Indeed, MEFs derived from transgenic mice overexpressing *HMGA1P7* show a higher growth rate, and lower susceptibility to senescence with respect to the WT counterpart. Moreover, flow cytometry showed an increase of cells in S phase as expected given the ability of HMGA1 to increase the E2F1 activity [45].

In contrast to a report that ectopic overexpression of *HMGA1* reduces the lifespan of IMR90 cells [46], *HMGA1P7-*MEFs that have more abundant HMGA1 protein levels, senesce later with respect to WT MEFs. In agreement with our findings, we obtained the opposite result in *Hmga1*-null MEFs [47]. It is likely that the cellular context influences the effect exerted by HMGA proteins on cell growth. Moreover, the different experimental approach, one *in vivo* and one *in vitro*, may account for these contradictory results. In fact, discrepancies between *in vitro* and transfection approaches were reported in a study of the p53 pathway [48]. The behaviour of the *Hmga1*-null and *HMGA1P7*-MEFs described here supports the oncogenic role of HMGA overexpression, which is a feature of malignant neoplasias. In conclusion, our finding that HMGA1P7-overexpressing MEFs grow faster and senesce later than their WT counterpart sustains our model in which *HMGA1Ps* act as ceRNAs that regulate *HMGA1* and other genes by competing for shared miRNAs thus contributing to cancer progression.

## MATERIALS AND METHODS

### Cell culture and transfections

HEK293, MCF7, 8505c, and MEF (from 12.5-day-old embryos) cells were maintained in DMEM supplemented with 10% foetal calf serum (GIBCO; Invitrogen), glutamine and antibiotics. Cells were regularly tested with MycoAlert (Lonza) to ascertain that cells were not infected with mycoplasma. Cells were transfected using Lipofectamine plus reagent (Invitrogen) according to the manufacturer's instructions. The transfected cells were selected in a medium containing geneticin (Sigma). For each transfection, several geneticin-resistant mass cell populations were isolated and expanded for further analysis. Transfection efficiency was verified for each experiment by evaluating GFP expression. To inhibit *HMGA1P6* and *HMGA1P7* expression, small interfering RNAs and corresponding scramble small interfering RNAs were designed and used as suggested by the manufacturer (RIBOXX).

### Human thyroid and ovary tissue samples

Neoplastic and normal human thyroid tissues were obtained from surgical specimens and immediately frozen in liquid nitrogen. Thyroid tumours were collected at the Service d'Anatomo-Pathologie, Centre Hospitalier Lyon Sud, Pierre Bénite, France. The tumour samples were frozen until required for RNA or protein extraction. We declare that informed consent for the scientific use of biological material was obtained from all patients. TissueScan Ovarian Cancer Tissue Real-time PCR Panel were purchased from Origene (HORT302).

### RNA extraction and quantitative reverse transcription PCR

Total RNA was extracted from tissues and cell cultures with Trizol (Gibco) according to the manufacturer's instructions. For mRNA detection, we reverse transcribed total RNA from cell lines by using the QuantiTect Reverse Transcription Kit (Qiagen), and then Real-time PCR was performed by using Power SYBR Green PCR Master Mix (Applied Biosystems) and the following primers:

*HMGA1*-Fw 5'-aaggggcagacccaaaaa-3' *HMGA1*-Rev 5'-tccagtcccagaaggaagc-3'
*HMGA1P6*-Fw 5'-gcagacccacaaaactgga-3' *HMGA1P6-*Rev 5'-gagcaaagctgtcccatcc-3'
*HMGA1P7*-Fw 5'-gctccttctcggctcctc-3' *HMGA1P7*-Rev 5'-gcttgggcctcttttatgg-3'
G6PD-Fw 5'-acagagtgagcccttcttcaa-3' G6PD-Rev 5'-ataggagttgcgggcaaag-3'
*Hmga1*-Fw 5'-ggcagacccaagaaactgg-3' *Hmga1*-Rev 5'-ggcactgcgagtggtgat-3'
*Ccna*-Fw 5'-cttggctgcaccaacagtaa-3' *Ccna*-Rev 5'-caaactcagttctcccaaaaaca-3'
*Ccnb*-Fw 5'-gcgctgaaaattcttgacaac-3' *Ccnb*-Rev 5'-ttcttagccaggtgctgcat-3'
*G6pd*-Fw 5'-cagcggcaactaaactcaga-3' *G6pd*-Rev 5'-ttccctcaggatcccacac-3'
*Epha3*-Fw 5'-tggctccttggacagtttct-3' *Epha3*-Rev 5'-ttcccacaagctccatgact-3'
*Hjurp*-Fw 5'-gagaactggccatcttgcag-3' *Hjurp*-Rev 5'-aaggtgtttccgggcact-3'
*Kif26b*-Fw 5'-aagaggcaggctctcaagc-3' *Kif26b*-Rev 5'-gcagagaaagcaagggtcctt-3'
*S1pr3*-Fw 5'-agatgcgccttgcagaac-3' *S1pr3*-Rev 5'-agagtggtggtgggttcct-3'
*Pde3B-*Fw 5*'-*ccttgtatttcccgagaacagat-3' *Pde3B-*Rev 5*'-*ggtaatgaggtttacaccactgc-3'
*Hmga2-*Fw 5*'-*aaggcagcaaaaacaagagc-3' *Hmga2-*Rev 5*'-*ttgtggccatttcctaggtc-3'
*Ezh2-*Fw 5*'-*tggaagcagcggaggata-3' *Ezh2-*Rev 5*'-*gtcactggtgactgaacactcc-3'
*Vegf-*Fw 5*'-*aaaaacgaaagcgcaagaaa-3' *Vegf-*Rev 5*'-*tttctccgctctgaacaagg-3'

The 2^−ΔΔCt^ formula was used to calculate the differential gene expression.

### Plasmids

For transfection of miRNA oligonucleotides, cells were transfected with 50 nmol/ml of miRNA precursors or with a control no-targeting scrambled oligonucleotides (Ambion, Austin, TX) using siPORT neoFX Transfection Agent (Ambion). For the *HMGA1P6* expression construct (pCAG-*HMGA1P6*) and the *HMGA1P6* luciferase reporter construct (pGL3-*HMGA1P6*), the entire sequence of *HMGA1P6* gene (ENST00000418454.1) was amplified by using the primers Fw *HMGA1P6* 5'-tcctctaattgggactccga-3' and Rev *HMGA1P6* 5'-ttactcagatcccaggcaga-3'. The amplified fragment was cloned into pCAG vector kindly given by Dr. S. Soddu, and into pGL3-Control firefly luciferase reporter vector (Promega), respectively. For the *HMGA1P7* construct (pCAG-*HMGA1P7*) and the *HMGA1P7* luciferase reporter construct (pGL3-*HMGA1P6*), the entire sequence of the *HMGA1P7* gene (ENST00000406908.1) was amplified by using the primers Fw *HMGA1P7* 5'-agccagtcgagctggaggtc-3' and Rev *HMGA1P7* 5'-ctgcaatgtgtactcagagc-3'. The amplified fragment was cloned as described for the *HMGA1P6* constructs. All the generated vectors were confirmed by sequencing. The Renilla luciferase vector (pRL-CMV), for transient transfection efficiency, was purchased from Promega. The 3' UTR region of the *HMGA1* gene has been previously described [34].

### Protein extraction, western blotting and antibodies

Protein extraction and Western blotting were performed as previously described [49]. The primary antibodies used were anti-EZH2 (AC22) and anti-Cyclin D2 (2924) from Cell Signaling; anti-Actin (sc-1615), anti-Vinculin (sc-7649), anti-γ-Tubulin (sc-17787), and anti-E2f1 (sc-193) from Santa Cruz Biotechnology; anti-VEGF (ab46154) from Abcam. Antibodies versus the HMGA1 and HMGA2 proteins are described elsewhere [50, 51]. Blots were visualized by using the Western blotting detection reagents (GE Healthcare).

### Cell migration and invasion assay

Cell migration and invasion experiments were performed as previously described [44].

### Dual-luciferase reporter assay

For dual-luciferase reporter assay, 3 × 10^5^ HEK293 cells were co-transfected in 6-well plates with the pGL3-*HMGA1P6* or the pGL3-*HMGA1P7* luciferase reporter vectors, together with the Renilla luciferase plasmid and miRNA precursors or a control no-targeting scrambled oligonucleotides (Ambion), using siPORT neoFX Transfection Agent (Ambion). The pRL-TK control vector expressing Renilla luciferase (Promega) was used for normalization of cell number and transfection efficiency. Luciferase activity was measured 48 hours after transfection using the Dual-Luciferase Reporter Assay System (Promega) with a Lumat LB 9507 apparatus (Berthold Technologies).

### Flow cytometric analysis

HEK293 cells were transfected with *HMGA1P6*, *HMGA1P7* and the empty vector, and analysed by flow cytometry after 48 hours of growth under normal culture conditions. Primary MEFs were obtained from 12.5-day-old embryos. The MEFs were minced and used to establish single cell suspensions and then analysed by flow cytometry after 48 hours of growth under normal culture conditions. Briefly, cells were harvested in PBS containing 2 mmol/l EDTA, washed once with PBS, and fixed for 2 hours in cold ethanol (70%). Fixed cells were washed once in PBS and treated with 40 μg/ml RNase A in PBS for 30 minutes. They were then washed once in PBS and stained with 50 μg/ml propidium iodide (Roche). Stained cells were analysed with a fluorescence activated cell sorter (FACS) Calibur (Becton-Dickinson), and the data were analysed using a mod-fit cell cycle analysis programme.

### Generation and genotyping of mutant mice

The 3.5 kb *HMGA1P7* of the pCAG-*HMGA1P7* expression plasmid was excised with *Sal*I & *Hind*III restriction endonucleases by cleaving 10 μg of the plasmid. The fragment was purified from SeaKem GTG agarose (avoiding exposure to UV light) using the Qbiogene Geneclean Spin kit, then dialysed 24 h against 2 l microinjection buffer (10 mM Tris.HCl pH 7.2, 0.1 mM EDTA), and diluted to a concentration of 4 ng/μl. The DNA was injected in three sessions into C57BL/6N-derived zygotes. For this purpose, C57BL/6N female mice (bred at PolyGene from parents obtained from Charles River) were superovulated at 28-34 days of age and mated in the PolyGene mouse facility to C57BL/6N breeder males, originally also obtained from Charles River. Injected zygotes were cultivated overnight and transferred into pseudopregnant B6CBAF1 females, also from Charles River. The animals were kept in individually ventilated cages. Injections were performed at the PolyGene labs in Rümlang, Switzerland. Pups were biopsied at weaning and analysed for transgene integration by PCR, using the PCR primer combination: Fw 5'-ggcatgtcccactctatt-3'; Rev 5'-caattcctgcaatgtgtactc-3'. All mice were maintained under standardized nonbarrier conditions in the Laboratory Animal Facility of the Istituto dei Tumori di Napoli (Naples, Italy), and all studies were conducted in accordance with Italian regulations for experimentations on animals.

### SA-β-gal assay

4 × 10^4^ MEFs, plated 24 hours before the assay, were washed twice with PBS and immersed in fixation buffer (2% [w/v] formaldehyde, 0.2% [w/v] glutaraldehyde in PBS) for 7 minutes. After 3 additional PBS washes, the cells were allowed to stain overnight in staining solution (40 mM citric acid/sodium phosphate, pH 6.0; 150 mM NaCl; 2.0 mM MgCl2; 1 mg/ml X-gal) at 37°C without CO2 to avoid changes in pH. The next day, the staining solution was replaced with PBS, and the stained and unstained cells were counted by light microscopy (at least 24 fields).

### Microarray analyses

RNAs extracted from *HMGA1P7* transgenic and WT MEFs (two biological replicates for each sample) were hybridized to the Affymetrix GeneChip Mouse Gene 2.0 ST oligonucleotide arrays. Hybridization, washing, staining, scanning, and data analysis were performed by the Affymetrix Microarray Unit at the IFOM-IEO campus, Milan, Italy, according to the manufacturer's instructions. Data were analyzed using Partek Genomics Suite version 6.6. Transcripts showing a significant fold change variation (p<0.05) were examined.

### Statistical analysis

Data were analyzed using a two-sided unpaired Student's t test (GraphPad Prism, GraphPad Software, Inc.). Values of P<0.05 were considered statistically significant. The mean +/− s.d. of three or more independent experiments is reported. Regression analyses and correlation coefficients were generated using GraphPad Prism, GraphPad Software, Inc.

## SUPPLEMENTARY FIGURE


