# Supplementary material for: HMGA1 pseudogenes as candidate proto-oncogenic competitive endogenous RNAs
Source: Oncotarget. 2014 Jul 15;5(18):8341–54. doi: 10.18632/oncotarget.2202 (PMC4226687; doi:10.18632/oncotarget.2202)
Supplement: Supplementary file 1 [file oncotarget-05-8341-s001.pdf]

SUPPLEMENTARY FIGURE

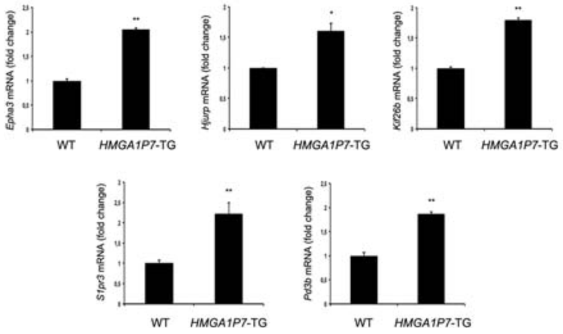

Supplementary Figure S1: Genes modulated by *HMGA1P7* expression. qRT-PCR validation of the Affymetrix GeneChip arrays hybridized with RNAs from WT and *HMGA1P7* overexpressing MEFs. The results are reported as the mean of values with error bars indicating SD (mean ± SD); n =3. *, $P < 0.05$ **, $P < 0.01$ (t test).
